# Mycobacterium abscessus urinary tract infection: case report

**DOI:** 10.1590/2175-8239-JBN-2018-0260

**Published:** 2019-05-30

**Authors:** Jadson Soares Laudelino, Flávio Teles Farias, André Falcão Pedrosa Costa, Vitorino Modesto Santos

**Affiliations:** 1 Universidade Estadual de Ciências da Saúde de Alagoas, Maceió, AL, Brasil.; 2 Hospital das Forças Armadas, Brasília, DF, Brasil.; 3 Universidade Católica de Brasília, Taguatinga, DF, Brasil.

**Keywords:** Mycobacterium Infections, Nontuberculous, Urinary Tract Infections, Tuberculosis, Infecções por Micobactéria não Tuberculosa, Infecções Urinárias, Tuberculose

## Abstract

Urinary tract infection is a serious public health issue that predominantly affects women. In men, it is more often associated with prostatic hyperplasia and bladder catheterization. Urogenital tuberculosis presents with nonspecific with nonspecific symptoms and the diagnosis can be made in the presence of sterile leukocyturia and recurrent infection with acid urine. Non-tuberculous mycobacteria or other non-tuberculosis mycobacteria are opportunistic pathogens that inhabit the soil, water or environment surfaces, and usually cause diseases in immunocompromised individuals. Mycobacterium abscessus is an agent that causes lung, skin and soft tissue hospital infections. Urinary tract infections by this pathogen are rare.

## Resumo

Infecção do trato urinário é um sério problema de saúde pública que acomete predominantemente as mulheres. Em homens, está mais relacionada com hiperplasia prostática e cateterismo vesical. A tuberculose urogenital cursa com sintomas inespecíficos e o diagnóstico pode ser aventado na presença de leucocitúria estéril, e infecção recorrente com urina ácida. Micobactérias não tuberculosas ou mycobacteria other than tuberculosis são patógenos oportunistas que habitam o solo, a água ou superfícies do meio ambiente, e geralmente causam doenças em imunodeprimidos. Mycobacterium abscessus é um agente que causa infecções nosocomiais, pulmonares, de pele e de tecidos moles. Infecção urinária decorrente desse patógeno é considerada rara.

## Introduction

Urinary tract infections (UTIs) are caused by several microorganisms, and the main pathogen involved is *Escherichia coli*, followed by *Klebsiella pneumoniae, Proteus mirabilis, Enterococcus faecali*s and *Staphylococcus saprophyticus*.^1^^,^ 2 Women are more often affected than men, a phenomenon explained by various factors - anatomical or behavioral; in men, prostatic hyperplasia and urinary instrumentation are factors that favor the occurrence of UTIs.

Urogenital tuberculosis (UGTBC) should be considered a differential diagnosis of UTIs; the clinical symptoms are nonspecific, the most common symptoms are dysuria, polaquiuria and back pain, and there may be pyuria and microscopic hematuria.^3^ The UGTBC is based on the growth of the pathogen in urine culture, sown in a specific medium and, rarely, on pathology findings.

Non-tuberculosis mycobacteria (NTM), are those that do not belong to the Mycobacterium tuberculosis (M. tuberculosis and M. bovis) complex, and M. leprae.^4^ More than 125 NTM species that cause diseases in humans have already been identified.^4^ These pathogens are usually isolated from soil and from natural or treated water sources.^4^ There is no evidence of human-animal or inter-human transmission, and the disease results from environmental exposure, although the source of infection is not usually identified.^4^ In developed countries, the incidence rate ranges from 1 to 2 cases per 100,000 inhabitants; however, since non-tuberculous mycobacterioses are not compulsorily notifiable diseases, their true incidence is unknown.^4^ Chronic lung infection is the most frequent clinical manifestation, accounting for 90% of the cases, followed by disseminated disease, lymph node infection and skin or soft tissue infection.^4^ We report a case of an adult man with urinary infection *by M. abscessus*, a microorganism that has been rarely described as an etiologic agent of UTI.

## Case report

**Figure 1:**
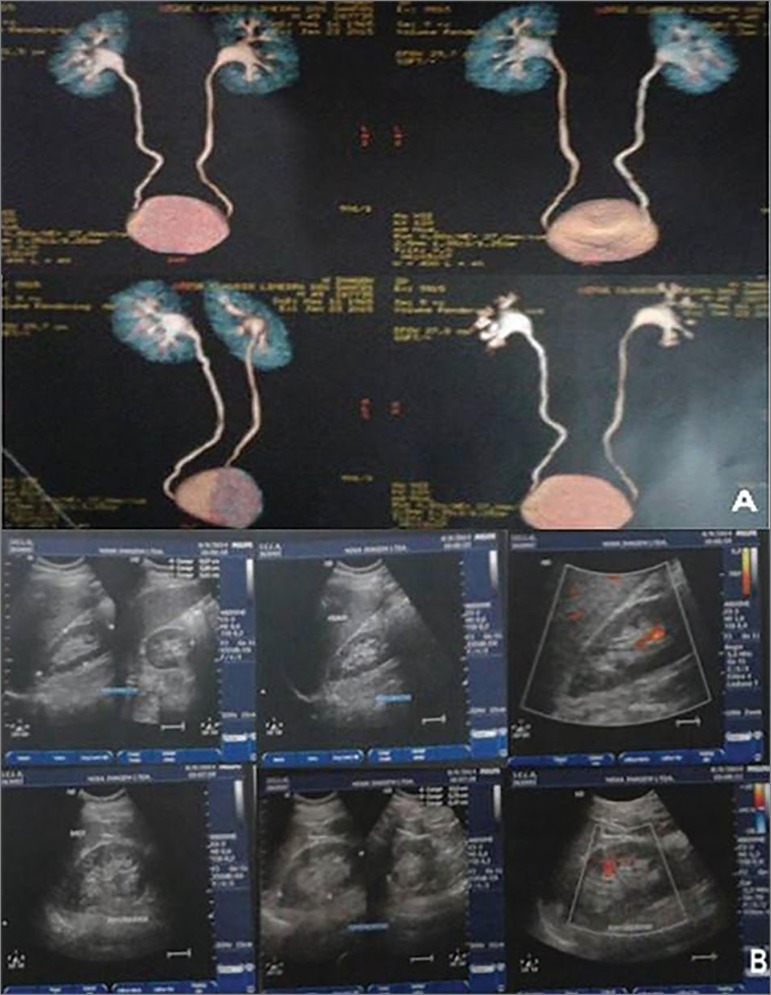
Abdominal CT (A) and Ultrasound (B) scan images showing mild renal pelvis ectasia and prostate calcifications.

50-year-old man, accountant, from a town in the countryside of Alagoas. He sought medical attention two years before a nephrological evaluation, complaining of pain in the right iliac and suprapubic fossa, nausea, vomiting and intense dysuria. He had no hematuria, fever, or weight loss. After consultation with an urologist, he used ciprofloxacin and tamsulosin for seven days. With no symptom improvement, the treatment regimen with ciprofloxacin was extended for another 14 days. The following exams were ordered in the investigation: urinary tract ultrasonography, prostate-specific antigen dosage and urodynamics, which results remained within the normality range. Urinalysis showed a pH of 7 and numerous pyocytes; and the erythrocyte sedimentation rate was 75 mm/1^st^ hour. Computed tomography and ultrasound images of the abdomen revealed discrete renal pelvic ectasia and prostate calcifications (Figures 1A and B).

The initial physical examination of the nephrological evaluation did not detect significant alterations besides discomfort to deep palpation in the hypogastric region. Inflammatory markers, uroculture and EAS were requested for control purposes. Inflammatory markers were altered, leukocyturia persisted in the EAS, and uroculture was repeatedly negative. In this way the possibility of *M. tuberculosis* urinary infection was raised and specific culture was requested for this infectious agent. Two months later, the patient returned for consultation complaining of persistent dysuria, bladder discomfort (pressure), voiding urgency and nocturia. The uroculture resulted positive for *M. abscessus*. In the Löwenstein-Jensen solidified medium, fast-growing mycobacteria formed acromogenic colonies with rough-to-smooth morphology. The biochemical tests revealed a reduction of nitrate (-), β-glycosidase (-), 5% tolerance to sodium chloride (+), sodium citrate (-), fructose fermentation (-), mannitol fermentation (-); picric acid (+) and Tween 80 hydrolysis (+).

We started treatment with clarithromycin (500 mg twice daily) and amikacin (15 mg/day), for 18 months. In the fifth month of treatment, the patient remained with urinary symptoms; however, in the tenth month he already showed important improvements. In the eighteenth month of outpatient follow-up, there was no clinical complaint and two urocultures for mycobacteria were negative.

## Discussion

Tuberculosis continues to represent a serious global health problem,^5^ and extra pulmonary involvement can occur in approximately 15% of the cases.^6^ UGTBC is the second site of this type of involvement and manifests as an insidious clinical picture with no specific symptoms, generally leading to late diagnoses.^7^ Diagnostic confirmation is established by finding *M. tuberculosis* in urine culture or by pathology examination of tissue samples.

The reported patient had UTI with many symptoms, and did not respond to treatment with the routinely used antibiotics. Considering the persistence of the symptoms in the presence of sterile uroculture, tissue calcifications and discrete anatomical alteration of the right pelvis, the hypothesis of UGTBC was proposed and uroculture was requested in a specific medium. The result was positive for *M. abscessus*, one of the nontuberculous mycobacteria that have very rarely been described as the etiology of UTIs.

NTMs are environmental opportunistic pathogens living in soil and drinking water systems.^8^ There are reports of hospital contamination, including epidemic outbreaks.^9^ There is no record of transmission from animals to humans, nor among humans.^4^ Shower water, soil material or pool water may be sources of infection.^10^ NTMs are believed to be acquired from the environment by ingestion, inhalation, or skin contact.^10^ Opportunistic infection is related to immunocompromised patients, such as those infected with HIV, individuals with chronic obstructive pulmonary disease, cystic fibrosis, and sequelae of pulmonary tuberculosis.^9^^,^ 11

Diagnosis is made by identifying the mycobacteria, taking into account phenotypic characteristics, biochemical tests and the production of carotenoid pigments, or through the necessary polymerase chain reaction in cases of persistent diagnostic uncertainties.^12^^,^ 13

Treatment is prolonged, it has adverse effects and a large number of patients are oligosymptomatic; therefore, the therapeutic decision should take into account the risk of disease progression, the type of NTM involved and the general patient's health.^4^ Therapy is considered successful when symptoms are eradicated or controlled, and control cultures are negative.^4^ Symptom improvement usually occurs after the fourth or sixth month of treatment and cultures are negative between the sixth and twelfth month.^4^ When this does not occur, treatment failure is considered.^4^ Drug therapy should be maintained for 12 months after negative cultures, and thus the average treatment time varies from 12 to 18 months.^4^

Regarding the therapeutic response, NTMs are categorized into two groups: easy to treat (the main representative is *M. kansasii*) and difficult to treat.^4^ The lung disease caused by *M. kansasii* is usually treated with rifampicin, ethambutol and isoniazid; aminoglycosides are associated in cases of severe or extensive lung involvement.^4^ Macrolides (clarithromycin and azithromycin) are used in difficult-to-treat NTMs.^4^ Fast-growing and difficult-to-treat bacteria (including M. fortuitum and M. abscessus) do not usually respond to drug therapy, and treatment should be based on drug sensitivity tests.^4^ Amikacin, clarithromycin, quinolones, doxycycline, imipenem, linezunide, sulfamethoxazole, and tigecycline should be tested.^4^ *M. abscessus* is a species of rapid growth and multidrug resistance that causes nosocomial infections.^14^^,^ 15 Pulmonary, skin and soft tissue infections are the main forms of clinical presentation of the disease.^16^ Contamination may occur after inoculation of the pathogen into skin lesions during small traumas or during surgical procedures.^17^ Disease outbreaks after invasive procedures have been described in the literature, related to different medical procedures: post-arthroscopy, laparoscopy and abdominoplasty.^14^ The main hypotheses proposed for these cases of infection are poor sterilization and the use of contaminated water to clean the equipment.^14^

In the treatment of infections caused by this agent, it is mandatory to remove foreign bodies such as prostheses, injured or necrotic tissue.^14^ The pathogen is sensitive to azithromycin, clarithromycin and amikacin.^14^ Studies with *in vitro* susceptibility tests of growth mycobacterial isolates promptly indicate that amikacin is the drug of choice, followed by fluoquinolones.^18^ For infections of the skin, soft tissue and bone caused by *M. abscessus*, the medicinal product should be clarithromycin (1,000 mg/day) or azithromycin (250 mg/day)) combined with a parenteral antibiotic (amikacin, imipenem or cefoxitin).^19^ In adults with normal renal function, amikacin is used at doses of 10 to 15 mg/day.^19^ Invasive procedures are indicated for extensive lesions and abscesses, or when there is no proper response from treatment.^19^

## Conclusions

In the present case, it was not possible to establish the contamination mechanism, even after extensive evaluation. Diagnosis was established after the hypothesis of infection by one of these germs based on sterile leukocyturia, which, when detected, should be included in the investigation of this rare pathology.
